# Effect of a biosynthetic bacterial 6-phytase on the digestibility of phosphorus and phytate in midlactating dairy cows

**DOI:** 10.1093/jas/skad032

**Published:** 2023-01-27

**Authors:** Yueming Dersjant-Li, Ivonne Kok, Edwin Westreicher-Kristen, Rubén García-González, Alessandro Mereu, Trine Christensen, Leon Marchal

**Affiliations:** Danisco Animal Nutrition and Health, IFF, Oegstgeest, The Netherlands; Schothorst Feed Research (SFR), Ruminants Group, Lelystad, The Netherlands; Schothorst Feed Research (SFR), Ruminants Group, Lelystad, The Netherlands; Danisco Animal Nutrition and Health, IFF, Oegstgeest, The Netherlands; Danisco Animal Nutrition and Health, IFF, Oegstgeest, The Netherlands; Danisco Animal Nutrition and Health, IFF, Brabrand, Denmark; Danisco Animal Nutrition and Health, IFF, Oegstgeest, The Netherlands; Animal Nutrition Group, Wageningen University and Research, Wageningen, The Netherlands

**Keywords:** dairy cows, digestibility, phytase, phosphorus, phytate

## Abstract

The effect of a biosynthetic bacterial 6-phytase (PhyG) on the digestibility and excretion of crude protein (CP), phosphorus (P), and phytate-P (PP) in midlactating dairy cows was investigated. Thirty Holstein-Friesians were assigned to three treatments with 10 cows per treatment in a randomized block design. Cows were fed forage (grass and corn silage) provided ad libitum, and a concentrate (without added inorganic phosphate) administered separately in amounts individualized per cow according to milk production, supplemented with phytase according to treatment. The formulated forage-to-concentrate-ratio was ~65%:35%. Dietary treatments comprised the control diet (CON) and CON supplemented with 2,000 (PhyG2,000) or 5,000 (PhyG5,000) phytase units (FTU)/kg DM in the total diet. The experiment comprised an 18-d preperiod for the collection of data to facilitate the allocation of cows to the treatments, followed by a 19-d experimental period comprising a 14-d diet adaptation period and 5 d of twice daily feces collection. Fecal samples were analyzed for the determination of apparent total tract digestibility (ATTD) of chemical constituents in the diet. The ATTD of PP was 92.6% in CON suggesting a high but incomplete degradation of phytate by ruminal microbial phytases. Cows fed PhyG2,000 exhibited increased ATTD of CP and PP [68.4% (2.7% points above CON) and 95.1% (2.5% points above CON), respectively] whilst PhyG5,000 further increased ATTD PP and also increased ATTD P [54.1% (7.8% points above CON)]; ATTD of Ca tended to be increased in PhyG5,000 vs. CON. Linear dose–response relationships were observed for ATTD of DM, CP, P, Ca, and PP. In addition, fecal excretion of P, and PP linearly reduced and that of Ca and CP tended to linearly reduce with increasing PhyG dose level. No difference was observed for DM intake and milk composition was unaffected except for milk protein which tended to be higher in cows fed PhyG5,000 than CON. In summary, the addition of exogenous phytase at 2,000 FTU/kg or higher to diets of lactating dairy cows improved P, PP, Ca, and CP digestibility and reduced fecal excretion of P, PP, and CP in a dose-dependent manner.

## Teaser

An exogenous phytase supplemented to a commercially relevant diet of low phosphorus content is found to be effective in improving phosphorus and protein digestibility and reducing their excretion in midlactating dairy cows. Dietary supplementation with phytase offers an important approach to optimizing nutrient balance and reducing environmental phosphorus and nitrogen pollution from dairy farms.

## Introduction

Ruminant livestock production accounts for up to 70% of the phosphorus (P) waste produced by farmed animals (Tamminga and Verstegen, 1992). In dairy farming, this waste arises mostly from the excretion of undigested P in feces. Excretion through feces (manure) results in P being returned directly to the soil, either through direct excretion (in pasture-based systems), or via application to nearby arable or grassland (in housed dairy farming systems). Both routes can result in the accumulation of P in the soil if practices are not carefully monitored and controlled. Excess P in the environment has a substantial negative impact on aquatic ecosystems with subsequent risks to human health and the economy (Sharpley et al., 1994; Withers et al., 2015). In some countries, such as the Netherlands, the use of manure is strictly regulated as part of efforts to reduce P accumulation in the environment. Here and in other regions worldwide, reducing P excretion from dairy cattle continues to be a major focus to minimize the negative impacts of excess P on the environment. Optimizing dietary P content so that P is fed closer to requirement levels and improving P utilization from feed are key strategies for reducing P excretion (Valk et al., 2000). Achieving this could lower feed costs by reducing the need to add costly inorganic phosphate from phosphate rock (a finite resource) if dietary P is more fully utilized. This will also increase the sustainability of dairy production, whilst allowing more flexibility in the choice of P-containing ingredients.

In plant-based feed ingredients, the majority of P is present in the form of phytate (salt of phytic acid (*myo*-inositol hexakisphosphate, IP_6_)). Phytate must be broken down to release inorganic phosphate (Pi) which can be used more directly by the animal for growth and maintenance. In monogastric animals that possess limited endogenous phytase and phosphatase enzyme activity, a wealth of research has shown that undigested phytate can act as a potent antinutrient due to its capacity to interact with mineral ions, proteins, and amino acids in specific regions of the digestive tract, reducing the accessibility and digestibility of these nutrients and impairing growth performance (Selle and Ravindran, 2007; Selle et al., 2012; Humer et al., 2015). As a direct result, monogastric diets are routinely supplemented with exogenous microbial phytase to improve P availability and utilization from the diet and reduce the antinutritional effect of phytate. Traditionally, it has been believed that phytase supplementation in dairy cattle diets is not warranted. This is due to the substantial phytase activity of ruminal bacteria that hydrolyze phytate, releasing Pi for later absorption in the small intestine (Raun et al., 1956; Yanke et al., 1998; Guyton et al., 2003). The seminal study of Morse et al. (1992) reported that phytate-P (PP) disappearance from a range of cereal and oilseed meal concentrates incubated with ruminal fluid was greater than 99% in vitro, and that apparent total tract digestibility (ATTD) of PP was 94% to 99% in lactating dairy cows. Similarly, Clark et al. (1986) reported ATTD of PP values higher than 95% in high producing dairy cows. However, more recent studies have reported lower and more variable estimates of ATTD of PP, of between 69% and 97% (Kinkaid et al. 2005; Brask-Pedersen et al. 2013; Jarrett et al. 2014). In practice, phytate degradability in the rumen may vary in relation to a variety of factors. These include the composition of the diet and forage-to-concentrate ratio that may alter the ruminal microflora and hence their capacity to digest phytate (Yanke et al., 1998; Humer and Zebeli, 2015), the ruminal P availability of individual ingredients (Haese et al., 2020) that may be influenced by processing methods designed to protect proteins from ruminal degradation (Bravo et al., 2000), and the faster flow rate of the digesta of modern high producing cows fed easily fermentable carbohydrate that may result in some PP leaving the rumen undegraded (Krämer et al., 2013; Humer and Zebeli, 2015). Furthermore, the neutral pH of the rumen that is typically pH 6.0 to 7.0 (Winter et al., 2015; Kim et al., 2018) may not be optimal for ruminal microbes producing cysteine phytases that exhibit optimum activity at pH 4.5 (Yanke et al., 1998; Puhl et al., 2008).

Exogenous microbial phytases, mostly of bacterial origin, are widely used as feed additives in commercial poultry and swine diets for improving P digestibility and utilization via their hydrolysis of phytate (Selle and Ravindran, 2007; Humer et al., 2015). Beneficial effects of exogenous phytase on the digestion and utilization of other nutrients including protein, amino acids (AA), energy, and starch are also evident in monogastric animals, especially in poultry (Selle et al., 2000; Ravindran et al., 2006; Truong et al., 2014, 2015). Literature on the effects of exogenous phytase on nutrient digestibility in lactating dairy cows is more limited but some studies have evaluated the potential for phytase to improve PP digestibility (Kincaid et al., 2005; Brask-Pedersen et al., 2013; Winter et al., 2015; Giagnoni et al., 2021).

Recently, a biosynthetic bacterial 6-phytase optimized for functionality in monogastric animals was developed and its characterization was published along with that of one if its biosynthetic variants (Christensen et al., 2020).

Against this background, the present study sought to investigate whether addition of this biosynthetic bacterial 6-phytase to diets of lactating dairy cows with no added Pi could increase the digestibility and utilization of P, PP, and CP. It was hypothesized that the addition of phytase to diets of dairy cows would improve the ATTD of P, PP, and CP.

## Materials and Methods

The study was carried out in accordance with the European Directive 2010/63 EU and the Dutch regulations for the care and use of animals in research. All experimental protocols and procedures were evaluated and approved by the Central Authority for Scientific Procedures on Animals (Centrale Commissie Dierproeven, Den Haag, the Netherlands) and by the Ethical Committee on Animal Experiments (Ethische Toetsing Dierproeven) of Schothorst Feed Research (Lelystad, the Netherlands). The approval code under which the trial was granted ethical approval was AVD246000202010084. The experimental work was completed during April and May 2021.

### Animals, diets, and feeding

The experiment was carried out with 30 Holstein-Friesian dairy cows at the experimental farm of Schothorst Feed Research BV (Lelystad, the Netherlands). Cows were multiparous and at the start of the experiment averaged 34 kg milk/d, 158 d in milk, 690-kg BW and 3.3 lactations. Cows were kept in a single group housed in a free stall barn equipped with cubicles (1.10 × 2.5 m) bedded with chopped straw, rubber floors and had free access to water. Cows were monitored for health daily and any signs of clinical disease were recorded and treated appropriately.

Treatment diets comprised of a control diet (CON) formulated without supplemental Pi and two experimental diets based on the CON diet but supplemented with a commercial phytase at a targeted dose level of 2,000 or 5,000 phytase units (FTU) per kilogram of total ration on a DM basis. The phytase was a biosynthetic bacterial 6-phytase, PhyG (Danisco Animal Nutrition and Health, IFF Inc., the Netherlands), expressed in *Trichoderma reesei*.

Forage and concentrates were fed to the cows separately. The formulated forage-to-concentrate-ratio was about 65%:35%. Cows had access to individual feed troughs accessed via Calan gates (American Calan, Northwood, NH) to measure the intake of the forage, and to a separate concentrate bucket located within the main feed through. The forage component comprised a mixture of a constant grass silage to corn silage ratio (30:70 on a DM basis) and was offered ad libitum. The chemical composition of the forages is shown in Table 1. Forages were mixed and provided to cows twice daily (at approximately 0700 and 1400 hours) via an automated forage dispenser system (Triomatic HP 2 300 hanging feeding robot) equipped with the Triomatic T40 feed kitchen with storage bunkers (Trioliet Feeding Technology, Oldenzaal, the Netherlands). Feed refusals were removed and weighed daily. The concentrates were manufactured by ABZ Diervoeding (Leusden, the Netherlands) and pelleted. The concentrates were supplied individually into the concentrate bucket of each cow, according to treatment, three times daily (at approximately 0500, 1230 and 1830 hours) via an automated concentrate dispenser (Hotraco, Hegelsom, the Netherlands) in amounts that were individualized per cow based on fat- and protein-corrected milk (FPCM)-yield. This was done in order to better account for the nutrient requirements of individual cows. The amounts of concentrate provided during the course of the experiment were not further modified because this may have affected milk production or DMI and confounded the ability to detect the effects of treatment on these response measures. Before producing the concentrates, ingredients were sampled for the analysis of PP and total P content. Afterwards, concentrates were optimized to contain a minimum of 2.3 g/kg of PP and a maximum of 3.1 g/kg of total P (equal to 2.8 g/kg DM on a total ration basis). Concentrates were formulated to contain phytate-rich ingredients with low ruminal degradability such as formaldehyde treated rapeseed meal and hydrothermal pressure-treated sunflower seed meal. For the estimation of fecal excretion, the external marker titanium dioxide (TiO_2_) was added to the concentrates, at a level of 7 g/kg (as is). The ingredients and chemical composition of the concentrates is given in Table 2, whilst the calculated chemical composition of the total rations is presented in Table 3. The total rations were formulated to meet nutrient requirements as recommended by the Dutch system (CVB, 2018) except for P that formulated at a level of 2.8 g/kg DM in the total ration and aimed to supply 90% of the recommended P requirement according to COMV (2005).

**Table 1.** Chemical composition of the forages

Item, g/kg dry matter (unless otherwise stated)	Grass silage	Corn silage
Dry matter g/kg	604	332
Ash	94	42
Crude protein	157	72
Crude fat	37	31
Neutral detergent fiber	511	349
Acid detergent fiber	295	211
Acid detergent lignin	28	23
Starch	nd	349
Sugar	105	14
Calcium	4.4	1.7
Phosphorus (P)	3.0	1.8
Phytate-P	0.0	0.0

nd, not determined.

**Table 2.** Ingredients, analyzed chemical composition, and phytase activity of the concentrates

Item	Concentrates^1^		
	CON	PhyG2,000	PhyG5,000
Ingredients, g/kg as fed			
Beetpulp	427	427	427
Sunflower meal	119	119	119
Rape seed meal	99.4	99.5	99.5
Wheat gluten meal	77.7	77.7	77.7
Corn	69.0	69.0	69.0
Oat hulls	66.5	66.5	66.5
Molasses beet	49.7	49.6	49.6
Corn gluten meal	19.9	19.9	19.9
Palm oil	18.1	18.1	18.1
Potato protein	13.5	13.5	13.5
Mineral premix^2^	12.4	12.4	12.4
Urea	9.94	9.94	9.94
Rumen-protected lysine	3.98	3.98	3.98
Magnesium oxide	3.09	3.09	3.09
Limestone	2.00	2.00	2.00
Salt	1.79	1.79	1.79
Titanium dioxide	7.00	7.00	7.00
Chemical composition, g/kg dry matter (unless otherwise stated)			
Dry matter, g/kg	901	901	902
Ash	81.0	82.1	82.0
Crude protein	269	269	268
Crude fat	46.6	46.6	46.6
Neutral detergent fiber	240	245	248
Starch	82.1	81.0	78.7
Sugar	155	161	159
Calcium	7.99	8.21	7.87
Phosphorus (P)	3.65	3.63	3.62
Phytate-P	2.33	2.11	2.00
Phytase, FTU/kg dry matter			
Total	431	6,181	21,725
Intrinsic	431	431	431
Exogenous	0	5,750	21,294

^1^CON, control; PhyG2,000, containing PhyG phytase at 2,000 FTU/kg DM; PhyG5,000, containing PhyG phytase at 5,000 FTU/kg DM.

^2^Chemical composition according to manufacturer in g/kg: 132 Ca, 1 P, 140 Mg, 75 Na, 116 Cl, 3 K, 0.9 S, 400 mg of Cu, 600 mg of Zn, 1600 mg of Mn 1,600 mg, 25 mg of Co, 110 mg of I, 30 mg of Se, 750,000 IU of vitamin A, 200,000 IU of vitamin D3, 1,500 IU of vitamin E.

**Table 3.** Chemical composition^1^, feed values and phytase activity of total dietary treatments

Item	Dietary treatment^2^		
	CON	PhyG2,000	PhyG5,000
Chemical composition, g/kg dry matter			
Dry matter, g/kg	474	470	469
Ash	65	65	66
Crude protein	155	152	150
Crude fat	35	35	35
Neutral detergent fiber	358	360	357
Acid detergent fiber	211	212	214
Acid detergent lignin	24	24	26
Starch	178	181	181
Sugar	73	72	73
Calcium	4.4	4.3	4.2
Phosphorus (P)	2.6	2.6	2.6
Phytate-P	0.7	0.7	0.6
Feed value^3^			
VEM/kg DM	980	978	978
DVE, g/kg DM	87	86	86
Phytase activity, FTU/kg dry matter			
Total phytase	136	1,949	6,532
Exogenous phytase	0	1,813	6,403

^1^Calculated from analyzed values presented in Tables 1 and 2, accounting for feed intake.

^2^CON, control; PhyG2,000, containing PhyG phytase at 2,000 FTU/kg DM ration; PhyG5,000, containing PhyG phytase at 5,000 FTU/kg DM ration.

^3^Feed value of diets estimated according to the Dutch evaluation system (CVB, 2018), where VEM is net energy for lactation, DVE is metabolizable protein.

### Experimental design

The experiment was carried out as a randomized block design with three dietary treatments and 10 blocks (replicates) per treatment. The experiment comprised an 18-d preperiod for the collection of data to facilitate the allocation of cows to the treatments, followed by a 19-d experimental period comprising a 14-d diet adaptation phase, as is recommended for digestibility trials (GfE, 1991), and 5 d of feces collection. During the preperiod, all cows were fed ad libitum with a mixture of grass silage and corn silage in the same proportions as mentioned above and were supplemented with concentrates (as described above but without supplemental phytase) based on FPCM production. Cows were allocated during the last 3 d of the preperiod to blocks based on parity, DMI, and FPCM production during the preceding week. Within blocks, animals were randomly allocated to treatments.

### Sampling, measurements, and chemical analyses

#### Milk

Cows were milked twice daily starting at 0400 and 1530 hours in a double-12 rapid exit milking parlor. Milk yield was individually recorded at each milking using calibrated electronic milk meters (DemaTron 70, GEA, Düsseldorf, Germany). Milk samples were collected per week from each cow on Monday evening, Tuesday morning, Wednesday evening, and Thursday morning until the end of the experimental period (day 19). Milk samples were preserved with a solution containing sodium azide and bronopol (0.3 mL of solution added per 50 mL of milk) and analyzed for fat, protein, lactose, urea, and somatic cell count by an accredited Dutch laboratory for monitoring milk quality (Qlip, Zutphen, the Netherlands), using Fourier transform infrared spectroscopy (MilkoScan FT6000/7, Foss Electric, Hillerød, Denmark). Two extra milk samples (50 mL) were taken per cow (morning and evening) during the last week of the experiment, pooled 1:1 (v:v) and analyzed for P content according to the spectrometric method ISO6491 (ISO, 1998).

#### Feed

The amounts of basal diet (forages) and concentrates offered and refused were individually weighed and recorded on a daily (forages) or weekly (concentrates) basis. The daily feed intake was calculated as the difference between the offered and refused amount for both basal diet and concentrates. Representative weekly samples of forages were collected, pooled per forage type by mixing equal amounts [on a fresh matter (FM) basis], and sent to the certificated laboratory Eurofins Agro NL (Wageningen, the Netherlands) for chemical analysis based on near infrared spectroscopy (NIRS). The concentrates were produced in one batch each, immediately sampled and analyzed. The chemical composition of these forages and concentrate samples was used to calculate the chemical composition of the total rations. During the final week of the experiment and 2 d prior to the start of fecal sampling, additional samples of the forages and concentrates were collected for the determination of the ATTD of chemical constituents. Forages were sampled daily and concentrates every 2 d. Samples were stored at –20 °C until later analysis. At the end of the experiment, forages were thawed at room temperature, pooled per type of forage by mixing equal amounts on a FM basis, freeze-dried for approximately 96 h in a Zirbus sublimator 3-4-5/20 (Zirbus Technology Benelux B. V., Tiel, the Netherlands) and ground to pass through a 1-mm screen using a Retsch ZM200 grinder (Retsch Benelux, Aartselaar, Belgium). The forage and concentrate samples were analyzed by Schothorst Feed Research (Lelystad, the Netherlands). The DM content was determined by drying at 103 °C to constant weight according to method ISO 6496 (ISO, 1998). Crude ash was determined gravimetrically after ashing the samples in a muffler furnace for 3 h at 550 °C, according to method ISO 5984 (ISO, 2002). The N content was determined by the Dumas method using a macro determinator (LECO CM928 MLC, LECO, Michigan, USA) according to method ISO 16634 (ISO, 2016), and the CP content was calculated as N × 6.25. The starch content (except in grass silage) was determined by the amylo-glucosidase method according to the procedures of Englyst et al. (1992), and sugar content was determined according to the Luff-Schoorl method. Crude fat (CFat) was determined by ether extraction after acid hydrolysis, according to method ISO 11085 (ISO, 2015). The NDF content was exclusive of residual ash and a heat-stable α-amylase was added during NDF extraction, according to ISO 16472 (ISO, 2006). The ADF content was exclusive of ash and determined according to ISO 13906 (ISO, 2008). The P content was determined based on the colorimetric method according to ISO 6491 (ISO, 1998) and contents of Ca and TiO_2_ were determined based on atomic absorption spectroscopy according to ISO 6869 (ISO, 2000). The content of PP in forages and concentrates was analyzed at Danisco Animal Nutrition Research Centre (Brabrand, Denmark) using the HPLC method described by Christensen et al. (2020) modified from Skoglund et al. (1998). Modifications to the analytical procedure were that the extraction of IP_6_ from the feces samples was carried out at a concentration of 0.20 g/mL using 1.0M HCl as solvent. The phytase activity in concentrate samples was analyzed by Danisco Animal Nutrition Research Centre (Brabrand, Denmark) according to a modified version of the 2000.12 AOAC method (Engelen et al., 2001). For this, one FTU was defined as the quantity of enzyme that released 1 µmol of inorganic orthophosphate from a 0.0051 mol/L sodium phytate substrate per minute at pH 5.5 at 37 °C.

#### Feces

Fecal grab samples (~500 g of fecal matter) were collected from all animals twice daily during the last 5 d of the experiment. Fecal sampling commenced at 0900 and 1300 hours on day 1, 3, and 5; and at 1100 and 1430 hours on days 2 and 4. This sampling pattern was applied to account for diurnal and day-to-day variations in marker excretion (Glindemann et al., 2009). Samples were immediately frozen at –20 °C and stored until later analysis. At the end of the experiment, fecal samples were thawed at room temperature, pooled per cow on an equal-weight (of FM) basis, freeze-dried for approximately 96 h in a Zirbus sublimator 3-4-5/20 (Zirbus Technology Benelux B. V., Tiel, the Netherlands), and ground to pass a 2-mm screen using a Retsch ZM200 grinder (Retsch Benelux, Aartselaar, Belgium). The content of moisture, CP (N × 6.25), NDF, starch, P, Ca, PP, and TiO_2_ was analyzed using the aforementioned methods.

#### Blood

Blood samples were taken from each cow from the coccygeal vein on day 4 of the collection period at approximately 1200 h. This time of day was selected as being between the timepoints of provision of fresh forage and of concentrate, therefore cows would have been consuming both components during the hours preceding sampling. Samples were analyzed for total (free) P, without a destruction step, according to method ISO 6491 (ISO, 1998). The serum cross-linked C-telopeptide of type I collagen (CTX), a marker for bone turnover, was analyzed according to a CTX-I ELISA method (IDS Plc., Tyne & Wear, UK).

#### BW and body condition score (BCS)

Individual BW and BCS were recorded twice daily directly after each milking. The BW was recorded via automatic weighing scale and the BCS was recorded using an automatic BCS system (DeLaval, Kansas City, MO). Body condition was scored based on the 1 to 5 scale method of Edmonson et al. (1989), where 1 = very thin and 5 = obese.

### Sample size, calculations, and statistical analysis

The sample size calculation was based on a two-sided test with a confidence level of 95% and a power of 0.80 to detect a statistically significant difference in ATTD of P in the phytase supplemented treatments compared to CON. The expected effect size was based on published data concerning the variance in P digestibility in lactating dairy cows (Valk et al., 2002; Wu et al., 2003; Kincaid et al., 2005; Knowlton et al. 2007). The chemical composition of the total rations was calculated based on the chemical composition and intakes of both forages and concentrates. The fecal excretion of DM was calculated for each cow from the daily TiO_2_ administration (g/animal) divided by the TiO_2_ concentration (g/kg DM) in feces. For this, a fecal recovery of TiO_2_ of 100% (Glindemann et al., 2009) was assumed. The fecal excretion of CP, starch, NDF, P, Ca, and PP was calculated as DM fecal excretion multiplied by the concentration of the respective chemical component in the feces. The ATTD of DM, CP, starch, NDF, P, Ca and PP was computed as ATTD (%) = [(intake – feces excretion)/intake]. For this calculation, the intake and feces excretion of each chemical component was in kilogram per day on a DM basis. The yield of FPCM (kg/d) was calculated on a 4% fat and 3.3% protein basis. The feed efficiency was calculated as FPCM divided by DMI both expressed in kg. Data on somatic cell count (SCC) were log transformed to obtain a normal distribution before statistical analysis. All data were averaged per cow and week for the statistical analysis.

All statistical analyses were performed using Genstat 18th edition (VSN International, Hemel Hempstead, UK). Data were analyzed by ANOVA to identify treatment effects. Treatment means comparisons were carried out using the Tukey test. Data are presented as least squares means and associated pooled SEM values. The statistical analyses for all variables (except P in milk and blood, and ATTD) were carried out using the data of the preperiod as a covariate, using the following model:


$$\begin{array}{l} Y_{ijk}=\mu +Block_{i}+Cov_{j}+T_{rtk}+{\;\varepsilon \;}_{ijk} \end{array}$$

where *Y*_*ijk*_ is the response variable, μ is the overall mean, Block_*i*_ is the effect of block (*i* = 1−10), Cov_*j*_ is the covariate (response during preperiod), *T*_*rtk*_ is the effect of dietary treatment (*k* = 1−3), and ε_*ijk*_ is the residual error.

For the statistical analysis of P content in milk and blood, and ATTD, the same model was used but without the preperiod as a covariate. In addition, the effect of phytase dose level on nutrient intake ATTD and fecal excretion of nutrients during the fecal collection period was analyzed by polynomial contrasts to determine the linear and quadratic response to increasing phytase dose, with consideration of uneven distribution between phytase dose levels. Statistical significance was declared at *P* < 0.05. 0.05 ≤ *P <* 0.1 was considered a tendency.

## Results

### Chemical composition of diets

In general, the chemical composition of the concentrates (Table 2) and total rations (Table 3) was similar among treatments. For the total rations, although the content of P was slightly lower than the targeted value (2.8 g/kg DM), the content among treatments was similar (2.6 g/kg DM for all). The PP level of the total rations was also slightly lower than formulated (–0.2 g/kg DM) but again was similar among treatments (0.6 to 0.7 g/kg DM). Phytase activity in the CON concentrate was low (431 FTU/kg, DM basis; Table 2). After deducting the activity in the CON diet from the total analyzed phytase activity in the concentrates of the PhyG2,000 and PhyG5,000 diets, the exogenous phytase activities in the total rations were calculated as 1,813 and 6,403 FTU/kg DM in PhyG2,000 and PhyG5,000, respectively (Table 3).

### Feed intake, milk yield and composition, and blood analysis

Table 4 shows the effect of PhyG supplementation on dry matter intake (DMI), BW, feed efficiency [expressed as DMI/FPCM (fat-and-protein-corrected-milk-production)], milk yield and composition of milk during the 19-d experimental period, and on blood analytes sampled on day 18. During this period, treatment had no effect on milk production or FPCM, that averaged 33.5 and 35.9 kg/d, respectively. Similarly, fat and lactose contents were unaffected by treatment. Milk protein content tended to be affected by dietary treatment (*P* = 0.08); cows fed PhyG5,000 tended to have a higher milk protein content than cows fed CON (3.72% vs. 3.68%, respectively). The P content of milk and of blood, and bone metabolism as indicated by CTX concentration, were unaffected by treatment. There was also no effect of treatment during the experimental period on DMI, BW, BCS, or feed efficiency.

**Table 4.** Effect of phytase supplementation on BW, dry matter intake, feed efficiency, milk production and composition (during the 19-d experimental period) and on blood analytes (measured on day 18)

Item	Dietary treatment^1^			SEM	*P*-value
	CON	PhyG2,000	PhyG5,000		
Dry matter intake, kg/d					
Forage	17.8	17.6	18.7	0.46	0.22
Concentrate	8.2	8.0	8.0	0.21	0.76
Total ration	26.0	25.4	26.7	0.40	0.10
Milk production					
Milk, kg/d	33.8	33.1	33.7	0.36	0.31
FPCM^2^, kg/d	36.1	35.5	36.0	0.45	0.55
Fat, g/d	1499	1471	1484	28.1	0.79
Protein, g/d	1228	1210	1243	15.1	0.33
Lactose, g/d	1522	1487	1520	17.6	0.31
Milk composition					
Fat, %	4.45	4.47	4.48	0.08	0.97
Protein, %	3.68	3.69	3.72	0.014	0.08
Lactose, %	4.49	4.50	4.49	0.013	0.97
Phosphorus, g/L	0.98	0.96	0.98	0.03	0.86
Urea, mg/dL	22.4	21.7	22.5	0.613	0.61
Somatic cell count, Log_10,_ cells/ml	1.7	1.7	1.6	0.049	0.32
BW, kg	692	688	689	3.13	0.61
Body condition score	3.11	3.08	3.07	0.02	0.43
Feed efficiency^3^	1.37	1.39	1.34	0.02	0.19
Blood analytes					
Phosphorus, mg/L	46.7	48.4	50.1	2.20	0.56
CTX, ng/mL^4^	1.95	1.53	1.66	0.24	0.46

^1^CON, control; PhyG2,000, containing PhyG phytase at 2,000 FTU/kg DM ration; PhyG5,000, containing PhyG phytase at 5,000 FTU/kg DM ration.

^2^Fat and protein corrected milk production (FPCM).

^3^Calculated as FPCM/DMI, both in kg.

^4^CTX, serum cross-linked C-telopeptide of type I collagen.

### Nutrient intake, excretion, and ATTD

The effect of treatment on dry matter intake (DMI) and nutrient intake, ATTD and fecal excretion during the 5-d fecal collection period (days 15 to 19) is presented in Table 5. During this period, there was no effect of treatment on DMI, intake of CP, starch, NDF, total P or Ca. However, intake of PP tended to be lower (*P* = 0.06) in Phy5,000 (16.5 g/d) than CON (18.7 g/d), but was similar in Phy2,000 (17.0 g/d).

**Table 5.** Effect of phytase supplementation on nutrient intake, fecal excretion, and apparent total tract digestibility (ATTD) during the 5-d fecal collection period

Item	Dietary treatment^1^			SEM	ANOVA *P*-value	Polynomial contrasts	
	CON	PhyG 2,000	PhyG 5,000			‘Linear’ *P*-value	‘Quadratic’ *P*-value
Intake							
Dry matter, kg/d	27.1	26.2	27.6	0.49	0.15	0.64	0.24
Crude protein, kg/d	3.97	3.83	4.00	0.70	0.26	0.86	0.36
Starch, kg/d	5.04	4.84	5.13	0.12	0.24	0.59	0.21
Neutral detergent fiber, kg/d	9.72	9.41	10.0	0.20	0.16	0.44	0.23
Phosphorus (P), g/d	69.9	67.3	70.6	1.16	0.15	0.80	0.29
Calcium, g/d	116	114	115	2.09	0.80	0.72	0.66
Phytate-P, g/d	18.7	17.0	16.5	0.63	0.06	0.24	0.40
Fecal excretion							
Dry matter, kg/d	8.19	7.43	7.58	0.22	0.06	0.16	0.25
Crude protein, kg/d	1.36^b^	1.21^a^	1.22^a^	0.03	0.01	0.08	0.21
Starch, g/d	126	120	105	8.30	0.23	0.15	0.58
Neutral detergent fiber, kg/d	4.18	3.74	4.02	0.13	0.08	0.40	0.20
Phosphorus (P), g/d	37.4^b^	33.6^a^	32.5^a^	1.11	0.02	0.01	0.34
Calcium, g/d	87.5^b^	86.5^b^	77.5^a^	2.62	0.04	0.06	0.93
Phytate-P, g/d	1.35^a^	0.87^b^	0.46^c^	0.095	<0.001	<0.001	0.04
ATTD, %							
Dry matter	69.9	71.6	72.7	0.76	0.06	0.01	0.49
Crude protein	65.7^a^	68.4^b^	69.5^b^	0.70	<0.01	<0.01	0.20
Starch	97.5	97.5	98.0	0.16	0.13	0.10	0.81
Neutral detergent fiber	57.1	60.2	59.9	1.29	0.21	0.11	0.20
Phosphorus (P)	46.3^a^	49.7^a,b^	54.1^b^	1.52	<0.01	<0.01	0.99
Calcium	24.1	23.3	33.3	2.85	0.05	0.02	0.37
Phytate-P	92.6^a^	95.1^b^	97.2^c^	0.45	<0.001	<0.001	0.13

^1^CON, control; PhyG2,000, containing PhyG phytase at 2,000 FTU/kg DM ration; PhyG5,000, containing PhyG phytase at 5,000 FTU/kg DM ration.

^a,b,c^Means bearing different superscript lower case letters within a row are significantly different at *P* < 0.05.

Dietary treatment affected the fecal excretion of CP (*P* = 0.01), total P (*P* = 0.02), PP (*P* < 0.001), and Ca (*P* = 0.04). Fecal excretion of CP, total P and PP was reduced in cows fed PhyG2,000 and PhyG5,000 compared with CON (by 10.3%, 13.1%, and 65.9%, respectively, in PhyG5,000 vs. CON). Fecal excretion of total P decreased linearly (*P* = 0.01) whilst that of PP decreased both linearly and quadratically with increasing PhyG dose level (*P* < 0.05) and that of CP tended to decrease linearly (*P* = 0.08). Fecal excretion of Ca was reduced (by 11.4%) in PhyG5,000 but not in PhyG2,000 compared to CON, and tended (*P* = 0.06) to reduce linearly with increasing PhyG dose level. Dietary treatment also tended to affect the fecal excretion of DM (*P* = 0.06) and NDF (*P* = 0.08), without a linear or quadratic dose–response effect.

The ATTD of CP, total P, and PP were all affected by treatment (*P* < 0.01) and treatment also tended to affect ATTD of DM (*P* = 0.06) and Ca (*P* = 0.05). The ATTD CP was higher for cows fed PhyG2,000 and PhyG5,000 than CON (+2.7% and +3.8% points, respectively; *P* < 0.05) whilst ATTD of P was higher in cows fed PhyG5,000 compared to CON (by 7.8% points; *P* < 0.05) and ATTD of Ca tended to be higher in cows fed PhyG5,000 compared to CON (by 9.2% points). There were positive linear relationships between PhyG dose level and ATTD of DM, CP, P, Ca, and PP (*P* < 0.05). The increase in ATTD of PP between 0 and 5,000 FTU/kg was 4.6% points.

### Discussion

An important prerequisite to determining whether an exogenous phytase will improve P digestibility is that the animals should be fed below the P requirement. To achieve this, the total rations were formulated to contain a low P content (without Pi in the concentrate) that represented approximately 90% of the total P requirement according to the Dutch guideline (COMV, 2005). The total rations contained P at 2.6 g/kg DM in all treatments.

The employed phytase dose levels (2,000 and 5,000 FTU/kg DM) were selected based on the dose levels of other exogenous microbial 6-phytases that have been reported in the literature to improve P digestibility in dairy cows (Brask-Pedersen et al., 2013; Winter et al. 2015) and consideration of the economic feasibility of including PhyG phytase in dairy cow diets. The analysis of the diets suggested that the level of phytase activity in the PhyG2,000 and PhyG,5000 diets was broadly consistent with the target dose levels, after deduction of the analyzed activity in the CON diet. The analyzed activity of phytase in the CON diet was assumed to have been supplied by the presence of intrinsic phytase in the cereal ingredients of the concentrate, as has been observed in other phytase studies (Dersjant-Li et al., 2020a; Velayudhan et al., 2021).

Cows exhibited a similar DMI among the treatments (both in the 5-d fecal collection period and over the entire 19-d experimental period), and because the chemical composition was similar between diets, the intake of all chemical components among treatments was also similar, except for PP. The PP intake was 12% lower with PhyG5,000, compared to the CON diet. This appears to have resulted from the combination of a lower PP content of the concentrate in PhyG5,000 than CON (2.33 vs. 2.00 g/kg, respectively, with the same basal diet), together with a lower-than-formulated concentrate-to-forage intake ratio in PhyG5,000 (30:70 in PhyG5,000, 32:68 in CON on a DM basis, compared to the formulated ratio of 35:65). A reduced PP intake could, in theory, have contributed to the observed differences in nutrient digestibility and excretion in the PhyG5,000 treatment compared to the control diet. However, the reduction in excretion of PP in this diet was much greater than the reduction in PP intake (PP excretion reduction was 66% greater than CON in PhyG5,000) suggesting that the observed increases in nutrient digestibility and reductions in nutrient excretion in PhyG5,000 were due to the activity of the added phytase and not the differences in PP intake.

In vivo, exogenous microbial phytase effects hydrolysis of phytate (IP_6_) by the stepwise dephosphorylation of phytate (IP_6_, that contains an inositol ring with six attached phosphate groups), to its lower inositol-phosphate esters (IP_5_, IP_4_, IP_3_, IP_2_, and IP_1_), at each step releasing a phosphate group which can be absorbed and used by the animal for maintenance and growth (Schlemmer et al., 2001; Greiner and Konietzny, 2011). The hydrolysis of phytate by microbial phytase has been shown consistently in studies of monogastric animals to have a direct improvement effect on P availability and digestibility (Torres-Pitarch et al. 2017, 2019; Dersjant-Li et al., 2020b) but it can also indirectly improve the availability and digestibility of other nutrients that associate with ­phytate in the digesta (the so called ‘extra-phosphoric’ effects of phytase), such as other minerals in addition to P, proteins, and AA (Dersjant-Li et al., 2015). In the present study, cows fed the PhyG5,000 diet exhibited a clear increase in ATTD of CP. The ATTD of CP was improved by approximately 3% points with the lowest dose of phytase (2,000 FTU/kg DM). Whilst the means comparison did not suggest there was a further increase with the higher dose of phytase (5,000 FTU/kg DM), the polynomial contrasts confirmed a dose–response relationship that was linear rather than quadratic in nature. With the phytase dosed at 5,000 FTU/kg DM, the ATTD CP was 4% points above that of cows fed the control diet. In monogastric animals (poultry and pigs), bacterial 6-phytases including PhyG have been shown to improve both AA and protein digestibility as well as protein utilization in a dose-dependent manner (Amerah et al., 2014; Adedokun et al., 2015; Dersjant-Li et al. 2022a, 2022b). The average improvement in ileal digestibility of total AA by PhyG dosed at 2,000 FTU/kg in broilers was recently estimated to be 4.3% units above a negative control diet in a modeling study that analyzed data from four separate trials (Dersjant-Li et al., 2022b). The noted improvement in ATTD of CP by PhyG at 2,000 FTU/kg DM in the present study is slightly lower but broadly comparable with this. Several possible mechanisms have been proposed for the positive effect of exogenous phytase on protein and AA digestibility in monogastric animals. These include: a reduction in the abundance of binary (protein-phytate) and ternary (protein-Ca-phytate) complexes in the distal gastrointestinal tract (GIT) due to the hydrolysis of PP by phytase in the proximal GIT; reduced endogenous amino acid flows as a result of the degradation of dietary phytate that otherwise increases endogenous losses of AA (Cowieson et al., 2004); and enhanced intestinal absorption of AA (Liu et al., 2008; Selle et al., 2012). The mode of action of phytase in improving AA and/or CP digestibility in dairy cows is not clear. It may be speculated that the phytase improved CP digestibility due to the reduced antinutritional effect of rumen-bypass phytate. In the present study, the increased ATTD of CP was accompanied by a reduced CP content of the feces (by 0.14 kg/d with PhyG dosed at 5,000 FTU/kg DM vs. control) which suggests better utilization of protein from the feed. This level of reduction in CP excretion would be of interest to producers on environmental grounds since the on-farm burdens of N from dairy cow production are increasingly regulated in several countries.

The observed tendency towards a higher content of milk protein in the highest phytase dose treatment compared to the control is consistent with the increased CP digestibility and suggests there was a higher availability of protein in the PhyG5,000 diet, due to the activity of the phytase. To the authors’ knowledge, previous studies have not observed an effect of exogenous phytase on milk protein content in dairy cows. It is hypothesized that the higher CP digestibility led to increased AA absorption and that this increased the availability of AA for milk protein synthesis; it is known that milk protein synthesis is highly reliant on the availability of AA, particularly of methionine, lysine, and histidine (Kim and Lee, 2021). Further studies are needed to evaluate the effect of exogenous phytase on milk protein content in dairy cows.

This experiment also showed a clear effect of phytase supplementation on the ATTD of P and PP. The ATTD of PP in the CON treatment (93%) was considerably below the 98% observed in the early study by Clark et al. (1986) and closer to the 90% total tract PP degradability reported by Brask-Pedersen et al. (2013). This adds further weight to the hypothesis that, contrary to past beliefs, the PP of feeds may not be fully utilized by dairy cows. This may be particularly the case when phytate-rich and rumen-protected ingredients are included. It is in such diets that exogenous phytase could have most benefit. In the present study, cows exhibited ATTD of PP increases (above CON) of 2.5% and 4.6% points with PhyG dosed at 1,813 and 6,403 FTU/kg DM, respectively (based on analyzed phytase activity). Jarrett et al. (2014) reported a numerical increase in total tract PP digestibility from 96.7% to 97.6% in diets supplemented with 1,500 FTU/kg DM of an unidentified phytase. On the other hand, Kincaid et al. (2005) observed increased total tract PP digestibility from 80% to 85% with 427 FTU/kg DM of an *Aspergillus niger* phytase, whilst Giagnoni et al. (2021) reported a 1.4% increase in the ATTD of PP with 6,000 FTU/kg DM of a histidine acid phosphatase expressed in *Aspergillus oryzae* (digestibility coefficient 0.981 with phytase vs. 0.967 without). Clearly, the physiological stage of the dairy cows, the dietary composition, basal PP digestibility level and phytase source could all impact the effect of exogenous phytase on ATTD of PP.

Regarding P digestibility, there was a linear increase in ATTD of P with increasing phytase dose that resulted in an improvement of 8% points above CON when the phytase was dosed at 5,000 FTU/kg DM. This level of improvement in ATTD of P is greater than that reported in other studies. In fact, Winter et al. (2015) observed no effect of a *Citrobacter braakii* phytase on ATTD of P in diets with low P content (2.6 g/kg DM, equal to the present study). Contrary, Kincaid et al. (2005) observed a tendency (*P* = 0.07) towards increased ATTD of P in diets supplemented with *A. niger* phytase (5.4% points and 3.7% points above a P-adequate diet in diets containing cottonseed and steam rolled barley, respectively). Interestingly, in diets containing between 3.2 and 3.4 g P/kg of DM [i.e. above the 2.6 g/kg DM present study and above the Dutch recommended P requirement for dairy cows producing 40 kg/d milk of 3.3 g P/kg of DM (COMV, 2005)], Giagnoni et al. (2021) observed a tendency (*P* = 0.06) towards an interaction between lactation stage (early vs. mid) and phytase supplementation (presence vs. absence). The authors observed that ATTD of P tended to increase with phytase in early-lactation but was unaffected in midlactation, compared to control diets. However, ATTD of PP was improved regardless of lactation stage. In the present study cows were in midlactation, therefore the increased ATTD of P by phytase is in contrast to the findings of Giagnoni et al. (2021). The different result may be related to the differing P content of the employed diets in the two studies which was below the requirement in the present study but at, or slightly above, the requirement of midlactating cows in the study by Giagnoni et al. The dose-dependent increases in ATTD of PP and P were accompanied by dose-dependent reductions in total PP and P fecal excretion in the phytase-supplemented treatments (compared to CON). A reduction of 4.9 g/day in P excretion with PhyG at 5,000 FTU/kg DM could contribute to reducing the on-farm burden of total P in a similar manner to that mentioned for N above.

It appears that the method of provision of the diet may influence the observed effect of exogenous phytase on PP intake and digestibility. In most of the existing dairy cow literature, diets were provided as a TMR, a factor that could activate the added phytase in the diet before ingestion because of direct contact with moisture mainly coming from the forage portion of the TMR. This has been reported by Brask-Pedersen et al. (2013). In the present study, the phytase was provided within the concentrate, separately from the forage. The moisture content of the pelleted concentrate was low (~ 99 g/kg on average). Therefore, activation of the phytase could be considered less probable but this possibility cannot be completely excluded. Indeed, the analyzed PP content in the concentrates was slightly lower in the phytase-containing treatments than the control concentrates. Whether phytase was activated in the concentrates and, if so, to what extent, remains unanswered and needs attention in future studies.

The inorganic P content of blood, when used alongside other indicators, may provide an indication of the P nutritional status of lactating dairy cows (Forar et al., 1982). Blood P content is known to be influenced by dietary P intake (Read et al., 1986). A higher digestibility of P in diets of low P content might therefore be expected to lead to increased P in the blood or bone, if there was increased P absorption in the small intestine. No effect of the phytase on blood P or CTX (a marker for bone turnover) was observed in the present study. However, this does not necessarily mean an absence of effect. The concentrates were supplied three times daily (at approximately 0500, 1230, and 1830 hours) while the blood samples were taken at 1200 hours. Thus, the blood samples were taken 7 h after the first feeding by which time any absorbed P in blood may have already been delivered to tissues. Further, whilst there was no statistically significant effect, it was noted that blood P was numerically increased and CTX was numerically decreased, which may be suggestive of an improved P availability in the phytase treatments compared to control. The percentage of P in milk was also unaffected by phytase addition in the present study, which could be linked to the short-term nature of the study. However, it is known that P intake has little effect on P in milk (Forar et al., 1982) which is more related to the protein content of the milk (milk contains protein-bound P). It appears that the tendency for milk protein to be increased in the phytase-supplemented cows was not sufficient to have any impact on milk P content in the present study. The study by Kincaid et al. (2005) similarly found no effect of exogenous phytase on the P content of milk.

In this study, phytase dosed at the higher level (5,000 FTU/kg DM) also tended to improve ATTD Ca (*P* = 0.05). In monogastric animals, a positive effect of phytase on Ca digestibility has been attributed to a reduction in the formation of Ca-phytate complexes in the GIT as a result of the degradation of phytate. These complexes otherwise precipitate and are not readily digested above pH 5.0 (in the small intestine where Ca is absorbed), thereby decreasing the availability of free Ca for absorption and utilization (Selle et al., 2009). As the specific gut regions of activity of PhyG in the dairy cow GIT have yet to be elucidated, it is unclear whether a similar mode of action could have led to the increased Ca digestibility in cows fed PhyG5,000, or alternatively whether it was linked to improved P digestibility and therefore a better balance of Ca to P at the sites of absorption, leading to greater absorption of Ca. Further research is needed on this aspect.

Little is currently known about the site(s) of exogenous microbial phytase activity in the cow digestive tract. The study by Brask-Pedersen et al. (2013) measured the concentration of IP_6_ and of lower IP-esters in digesta from the rumen, duodenum, ileum, and feces and reported that degradation of phytate (IP_6_) occurred predominantly (although not exclusively) before the duodenum. The researchers also reported a dose-dependent increase in rumen IP_6_ degradation in the treatments containing exogenous phytase (up to a maximum of 9.9% points with phytase at 6,367 FTU/kg DM) which implies activity in the rumen. However, it was not possible for these authors to distinguish between phytate degradation occurring in the rumen and the TMR because of substantial (measured) pre-ingestion activity of the phytase. The active site of the PhyG phytase on phytate degradation in the dairy cow needs to be further evaluated. What is clear from the present results is that the phytase, wherever it was active, led to a dose-dependent increase in ATTD of PP compared to the unsupplemented control diet.

## Conclusions

The ATTD of CP, P, PP, and Ca were improved by dietary supplementation of low P diets with a biosynthetic bacterial 6-phytase in a linear dose-dependent manner; improvements above the control, unsupplemented diet, were significant at a phytase dose level of 2,000 FTU/kg for ATTD PP and CP and 5,000 FTU/kg for ATTD P. There was also a tendency for ATTD Ca to be improved by 5,000 FTU/kg. In addition, fecal P, PP, and CP excretion were reduced by 2,000 or 5,000 FTU/kg of the phytase, and milk protein content tended to be increased by 5,000 FTU/kg. This study has clearly shown that supplementation of the novel phytase at a dose level of 2,000 FTU/kg or higher to dairy cow diets offers potential as an approach for reducing fecal excretion of P and CP by improving their digestibility in the diet. In this way, phytase supplementation offers an important approach to optimizing nutrient balance and reducing environmental P and N pollution from dairy farms. Further research is recommended under long-term conditions and with early lactating dairy cows.

## Abbreviations

AA: amino acids
ADF: acid detergent fiber
ADL: Acid detergent lignin
ATTD: apparent total tract digestibility
BCS: body condition score
CON: control diet
CP: crude protein
CTX: serum cross-linked C-telopeptide of type I collagen
DMI: dry matter intake
FM: fresh matter
FPCM: Fat- and protein-corrected milk
FTU: phytase units
GIT: gastrointestinal tract
IP_6_: inositol hexakisphosphate
MCP: monocalcium phosphate
NDF: neutral detergent fiber
NIRS: near infrared spectroscopy
PhyG: biosynthetic bacterial 6-phytase
Pi: inorganic phosphate
PP: phytate phosphorus
SCC: somatic cell count
